# Early remission of deliberate self-harm predicts emotion regulation capacity in adulthood: 12.4 years follow-up of a randomized controlled trial of adolescents with repeated self-harm and borderline features

**DOI:** 10.1007/s00787-024-02602-8

**Published:** 2024-10-29

**Authors:** Iselin Solerød Dibaj, Anita Johanna Tørmoen, Ole Klungsøyr, Katharina Teresa Enehaug Morken, Egil Haga, Kine Johansen Dymbe, Lars Mehlum

**Affiliations:** 1 Sognsvannsveien 21, 0372 Oslo, Norway; 2 Årstadveien 17, 5009 Bergen, Norway

**Keywords:** Emotion regulation, Self-harm, Adolescence, DBT, Coping strategies

## Abstract

Emotion regulation capacity, critical for adult functioning and mental health, develops strongly during adolescence in healthy individuals. Deficits in emotion regulation is often referred to as emotion dysregulation [ED] and is associated with various mental health problems, including repeated deliberate self-harm [DSH] which peaks in adolescence. Dialectical Behaviour Therapy for adolescents [DBT-A] systematically targets ED through strategies such as changing coping behaviours and has previously been shown to effectively induce DSH remission in adolescents. However, whether such remission is associated with improved emotion regulation capacity in adulthood, and whether this effect is mediated by changes in use of coping strategies has not been previously studied. Prospective long-term follow-up study of an RCT comparing DBT-A with enhanced usual care [EUC] for adolescents presenting to community child and adolescent psychiatric outpatient clinics with borderline personality features and repeated self-harm. Assessments included both structured interviews and self-report at baseline and 1.6, 3.1 and 12.4 years follow-up. In the final follow-up, adult ED was measured and data were collected for 61 (80%) of the original 77 participants. DSH remission was assessed at 1.6 years follow-up, and use of coping strategies at 3.1 and 12.4 years follow-ups. A mediation analysis was conducted within a causal inference framework. Both treatment groups increased their use of functional coping skills from adolescence to adulthood, while only DBT-A was associated with decreases in dysfunctional coping. There was a direct effect of DSH remission 1 year after treatment on adult ED, particularly for participants who did not receive DBT-A. There was a negative association between reductions in dysfunctional coping and adult ED, however this did not mediate the effect of DSH remission. This is the first study to report that early DSH remission in adolescence predicted lower ED in adulthood. These results highlight the importance of early DSH remission and provides new insight into the long-term relationship between DSH and ED. Clinical trial registration information: “Treatment for Adolescents with Deliberate Self-harm”; http://ClinicalTrials.gov/;NCT00675129

## Introduction

Emotion dysregulation [ED], commonly referring to “patterns of emotional experience or expression that interfere with goal-directed activity” [1], is associated with increased risks of deliberate self-harm [DSH; nonfatal self-injury with or without suicidal intent [2] and lower quality of life and functioning in adulthood [3]. ED is also associated with severe psychopathology, notably Borderline Personality Disorder (BPD) [4] where it is a core feature as well as assumedly an underlying factor in repetitive DSH [5]. At the same time, DSH and other dysfunctional coping strategies comprise behavioural patterns assumed to maintain ED through positive and negative reinforcement [6, 7]. Conversely, adaptive emotion regulation [ER], defined as “the ability to flexibly regulate emotions according to contextual demands” [8], is negatively associated with DSH and BPD [9, 10] in adults, and improved ER has been found to mediate treatment effects on DSH in adults [10, 11]. Nevertheless, as ER capacity is still under development during adolescence, increased efforts have been made to study interventions that target DSH through enhanced ER in adolescents [12–15].

Dialectical Behaviour Therapy for adolescents [DBT-A], currently the treatment with the strongest evidence of effectiveness in the treatment of adolescents presenting with DSH and ED [16], helps patients replace DSH and dysfunctional coping strategies with more functional behaviour [17]. Compared to active control conditions, DBT-A has been associated with earlier DSH reduction [18], DSH remission [15], and lower DSH mean levels post treatment that was maintained 3 years later [14, 19]. One study found that improved ED mediated the effect of DBT-A on DSH remission [50], and adult trials have demonstrated that increased overall use of functional coping strategies mediated the effect of DBT on DSH [11] and BPD [20]. Some studies have identified specific coping strategies as particularly important in this context, e.g., “acceptance without judgment” [21, 22].

According to Linehan’s *skills deficit model* [10], ED is related to an inability to access functional coping strategies. In the absence of coping skills, behaviours such as DSH, though in most aspects highly dysfunctional, may be employed as attempts at regulating emotions [24], a notion supported by studies showing associations between ED and DSH in adolescents [12, 25]. More specifically, a recent meta-analysis found that negative affect increased shortly before DSH, and decreased shortly afterwards [26], however DSH has also been found to predict higher negative affect one hour later [27]. Selby and Joiner [28] argue that both DSH and other dysfunctional coping behaviour reinforces ED in the long-term through short-term reductions of negative affect which subsequently increases, thus creating a vicious circle. DBT-A gives high priority to early DSH reduction while not neglecting to address other dysfunctional ER strategies such as substance misuse [29], binge eating [30], self-criticism or blaming others [31].

Although DBT-A is considered effective, little is known about how or why it works for adolescents with ED [32], and studies of mechanisms of change are still sparse in this group [33]. In this study, we address this knowledge gap by examining treatment effects on an outcome related to the intervention’s “theory of change” [34], i.e. reducing the potentially reinforcing effect of DSH and dysfunctional coping on ED [35] in an important developmental phase. Few studies have reported on *dysfunctional* coping, however, a recent uncontrolled DBT trial including adults with BPD features found that reduced dysfunctional coping predicted improved ED, while increased functional coping did not [36]. In a process-outcome study, Kramer et al. [37] found that reductions in behavioural coping in early phases of psychotherapy mediated the short-term treatment effect in a sample of adult BPD patients.

Linehan [10] postulated that biological vulnerability combined with adverse life events such as an invalidating environment underlies ED development in young people that subsequently might transition into BPD. ER capacity has been shown to negatively moderate the relationship between early adversity and subsequent psychopathology [38]. Furthermore, trauma exposure is commonly observed in BPD [39], and recent trauma exposure might affect ED independently of coping behaviour [40]. Nevertheless, as ER capacity is still under development during adolescence, early intervention that targets ED might reduce risk of psychopathology in adulthood [41]. Although DSH naturally decreases with age, delayed or lacking DSH remission implies potentially years in which ED is reinforced [18]. To our knowledge, there is limited knowledge on the long-term effects of continuing with dysfunctional behavioural patterns such as DSH on adult ED.

This study aimed to investigate whether adult ED was influenced by early established behavioral patterns, such as persistent DSH and use of dysfunctional coping. We hypothesized that in a developmental perspective, successful treatment in adolescence might enhance emotion regulation capacity in adulthood if behavioural change is achieved. In that endeavour, we investigated whether DSH remission in adolescence had a direct effect on adult ED, and if this effect was mediated through subsequent reductions in dysfunctional coping.

## Methods

### Participants and procedure

For details regarding methods in the original RCT, see Mehlum et al. [14]. Participants were recruited from five child and adolescent psychiatric outpatient clinics in Oslo. New referrals were screened for current self-harm, and eligible participants and their parents were invited to further assessment where the remaining inclusion criteria were assessed. Inclusion criteria were ≥ 2 episodes of self-harm, wherein at least one had to be within the last 16 weeks, ≥ 2 criteria of DSM-IV BPD (*plus* the self-destructive criterion) *or* ≥ 1 criterion of DSM-IV BPD *plus* ≥ 2 subthreshold-lever criteria *plus* the self-destructive criterion. Patients who met inclusion criteria were further invited for baseline assessment, and randomized if they did not meet exclusion criteria or consent to study participation. Participants were excluded if they met diagnostic criteria for Bipolar I, any psychotic disorder, intellectual disability or Asperger’s syndrome, or were not fluent in Norwegian. In total, 294 patients underwent screening for eligibility, 97 advanced to clinical assessment and the final sample consisted of 77 adolescents aged 12–18 years. Subsequently, they were randomized to receive 19 weeks of DBT-A or EUC, stratified on gender, ongoing major depressive disorder and suicide intent in the most severe DSH episode within the last 16 weeks before baseline. The original RCT was powered to detect treatment effects on mean DSH frequency and initial calculations estimated that a sample size of 80 would be needed for 80% power with 5% error levels in a two-tailed test. Power and sample size calculations for mediation analysis is still somewhat limited, but there has been some indications that power to detect direct and indirect effects is higher and lower than that for total effects, respectively [42]. The randomization and allocation to treatment condition was performed by a research coordinator, who also administered blinding of independent assessors. Randomization was block-stratified with one undisclosed and variable blocking factor and used a concealment key. During the trial, management of the randomization was conducted by an external group. Therapists and assessors did not share work spaces and all assessors were blinded to treatment allocation. The mean time between randomization and post-treatment assessments was 31 weeks. Before the trial, 15 therapists enrolled in DBT training, and 8 of these completed a consistently adherent case according to the DBT global rating scale (adherence score > 4, range 0–5) and was subsequently included as DBT trial therapists. During the trial, DBT-A was delivered conforming to the manual developed by Miller et al. [17], and adherence ratings were evaluated as acceptable based on videotaped sessions (2 first sessions and 3 random) that were evaluated by an independent rater [14]. DBT-A emphasizes skills training to help patients reduce ED and includes both individual and group sessions. EUC was standard treatment as usual, mainly psychodynamic or cognitive behavioural therapy, sometimes in combination with psychopharmacological treatment. Naturally, EUC was not subjected to adherence ratings, and EUC therapists were allowed to extend treatment beyond the trial time if needed. The enhanced aspect of the treatment was that EUC therapists agreed to deliver at least weekly individual therapy sessions for the duration of the trial. In addition, all therapists received information on results from trial assessments made of their patients as well as training in suicide risk assessment and management before inclusion of patients commenced.

**Fig. 1:**
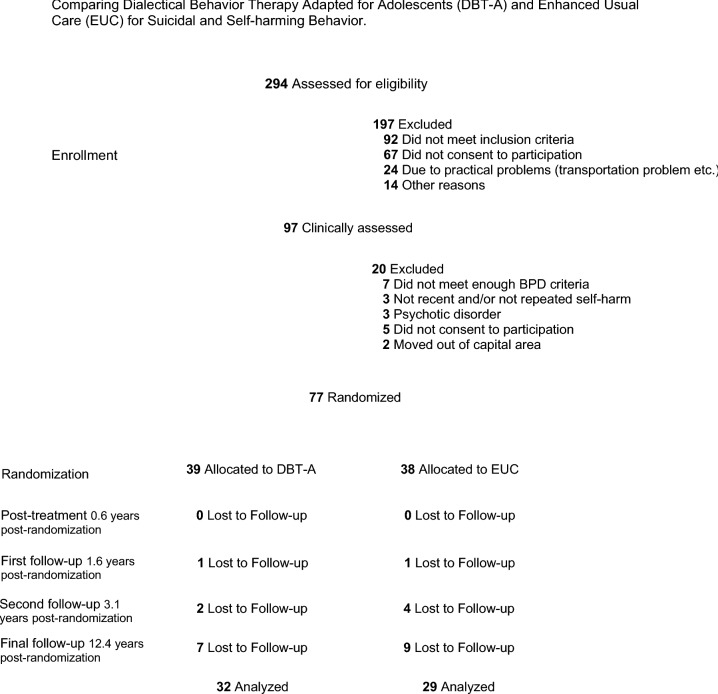
Flowchart (CONSORT) of participants in long-term follow-up of RCT comparing dialectical behaviour therapy adapted for adolescents (DBT-A) and enhanced usual care (EUC) for suicidal and self-harming behaviour

Consistently, the participation rate has been high in this study (see CONSORT diagram, Fig. 1). At the 1.6 year follow-up 75 members of the original sample participated (98%), and two declined further participation. At the 3.1 year follow up, 71 (92%) participated, three declined and two did not answer. Of these, 70 participants gave consent that we could contact them again for a long-term follow-up, where 61 participants were included (80%). We were unable to find correct contact information for one participant, four did not answer, two declined participation and two did not show up for the scheduled interview. The results of the primary analyses (DSH, suicidal ideation and depressive symptoms) are published elsewhere (Mehlum et al., under review). In total, 32 participants from the DBT-A group and 29 from the EUC group participated in the final follow-up assessment. Of the 90% who provided consent to be contacted for the final follow-up study, no participants had died, neither in suicide nor due to other causes. One participant did not complete the self-report questionnaires.

### Assessment and variables

Participants were assessed at multiple time points (both interview and self-report); in this analysis we have used data from baseline assessment as well as at follow-ups with a mean of 1.6, 3.1 and 12.4 years post-randomization. Interviewers were blinded to treatment condition and new interviewers were recruited for each follow-up point. All interviewers were clinicians that were trained in the specific assessment tools. Baseline assessments were conducted by two child and adolescent psychiatrists and two doctoral level clinicians. Follow-up assessments were made by independent interviewers; three child and adolescent psychiatrists and one psychiatrist at the 1.6 and 3.1 year follow-up and by one clinical psychologist at the 12.4 year follow-up. All interviews were recorded and a random selection of these, stratified on both treatment condition and time of inclusion, were tested for interrater-reliability [for details; see^14^, ^19^, ^43^.

ED was measured with the 16-item *Difficulties of Emotion Regulation Scale* [DERS-16; 12], a brief version of the extensively applied DERS, which construct validity has been supported through a large number of studies and been found to be sensitive to change in DBT and other treatments. Both the internal consistency and test–retest reliability of the DERS-16 is excellent, and it has been found to have high convergent validity with the full DERS [12, 44]. It has since its development been used as an outcome and mediator in clinical trials in different age groups and countries [45, 46] The *DBT Ways of coping checklist* [DBT-WCCL; 47] measured use of functional and dysfunctional coping strategies. The DBT-WCCL was developed to measure use of DBT skills and is an adapted version of the Revised Ways of Coping Check List [48] with good criterion validity. The functional and dysfunctional subscales have excellent and good internal consistency, respectively [47]. The DBT-WCCL has been used in DBT trials for both adults [49] and adolescents [50]. DSH was measured with the Lifetime Parasuicide Count [LPC; 51, which is a structured interview where participants are informed of the DSH definition and asked to report number of episodes for different types of self-harm methods (cutting, intox, burning, choking/hanging, jumping from a high place, gunshot, swallowed non-edible object, asphyxia, drowning, pierced skin, banged head/beat oneself and “other”). DSH remission was dichotomized and operationalized as one year without any DSH episodes after treatment. BPD was evaluated through the *SCID-II* for DSM-IV [52] and dichotomized. Traumatic experiences were recorded through the CARE interview [53] tapping exposure to various, specific traumatic experiences grouped into the categories “physical abuse”, “sexual abuse” and “other trauma”.

#### Data analysis

All statistical analyses were performed in STATA 17.0, including the ‘paramed’ package [54]. For the comparisons of ED and coping strategy use within and between the treatment groups, t-tests were performed. The mediation analysis was performed within the counterfactual framework of causal inference [55] where effects are formulated as *potential outcomes* [56]*.* First, we fitted linear regression models for the outcome (a function of the exposure, the mediator, the exposure-mediator interaction and the covariates) and for the mediator (a function of only the exposure and covariates). In the present analysis, we were interested in the effect of DSH remission [exposure] on adult ED [outcome], (not) through reductions in dysfunctional coping [mediator].

In practice, causal mediation analysis (CMA) mainly differs from the widely used product method [57] in terms of assumptions and that it allows for exposure-mediator interaction. In the absence of such interaction, the results will be identical in the two methods, however in the presence of such interaction the product method would produce biased estimates [55]. CMA assumes that there should be no unmeasured confounders on the association between the exposure and the outcome or the mediator, between the mediator and the outcome and that eventual confounders of the latter association cannot be affected by the exposure. Thus, it is important to control for confounding variables both between the exposure and outcome, between the exposure and mediator, between the mediator and the outcome as well as assume that confounders of the mediator outcome relationship are not affected by the exposure. Hence, treatment condition, BPD diagnosis at baseline and recent traumatic exposure were included, and none of these were assumed to be affected by DSH remission. As the data came from an RCT with a known effect of DBT on DSH, treatment condition was included in the model. In a randomized design, it is most pertinent to control for confounders between the mediator and outcome, since participants are not randomized on mediator values. Because our sample was not stratified on BPD, however consisted of adolescents with both a BPD diagnosis as well as subthreshold BPD, we assumed that having a full BPD diagnosis at baseline could potentially affect dysfunctional coping and long-term ED. Finally, we considered that recent trauma exposure was a factor that could potentially increase ED independently of DSH remission and use of dysfunctional coping skills, thus included it as a potential confounder.

DSH was dichotomized so that each participant had either T = 0 – no remission – or T = 1 – remission. Remission was operationalized as no DSH episodes within the last year. We calculated both direct and indirect effect estimates; where each of them can be interpreted as a causal contrast; either between the outcome for the two treatment groups with mediator being held fixed (direct effects, or between the outcome for two levels of the mediator while the treatment group is held fixed (indirect effects). The controlled direct effect is a direct effect where the mediator is set to a fixed level (in this case, the mean level = 0.12). Moreover, pure and total natural direct effects were calculated, which refers to direct effects wherein the mediator is set to the value it would “naturally” have given the level of exposure (in this case, either given DSH remission or lack thereof). The pure and total natural *indirect effects* refer to the values of the outcome corresponding to the mediator value in which participants either have (T = 1) DSH remission or not (T = 0). We calculated both marginal and conditional (on treatment condition) estimates, to differentiate average effects and conditional effects; i.e. whether the effect pathways could differ between participants who received DBT-A vs EUC.

**Fig. 2:**
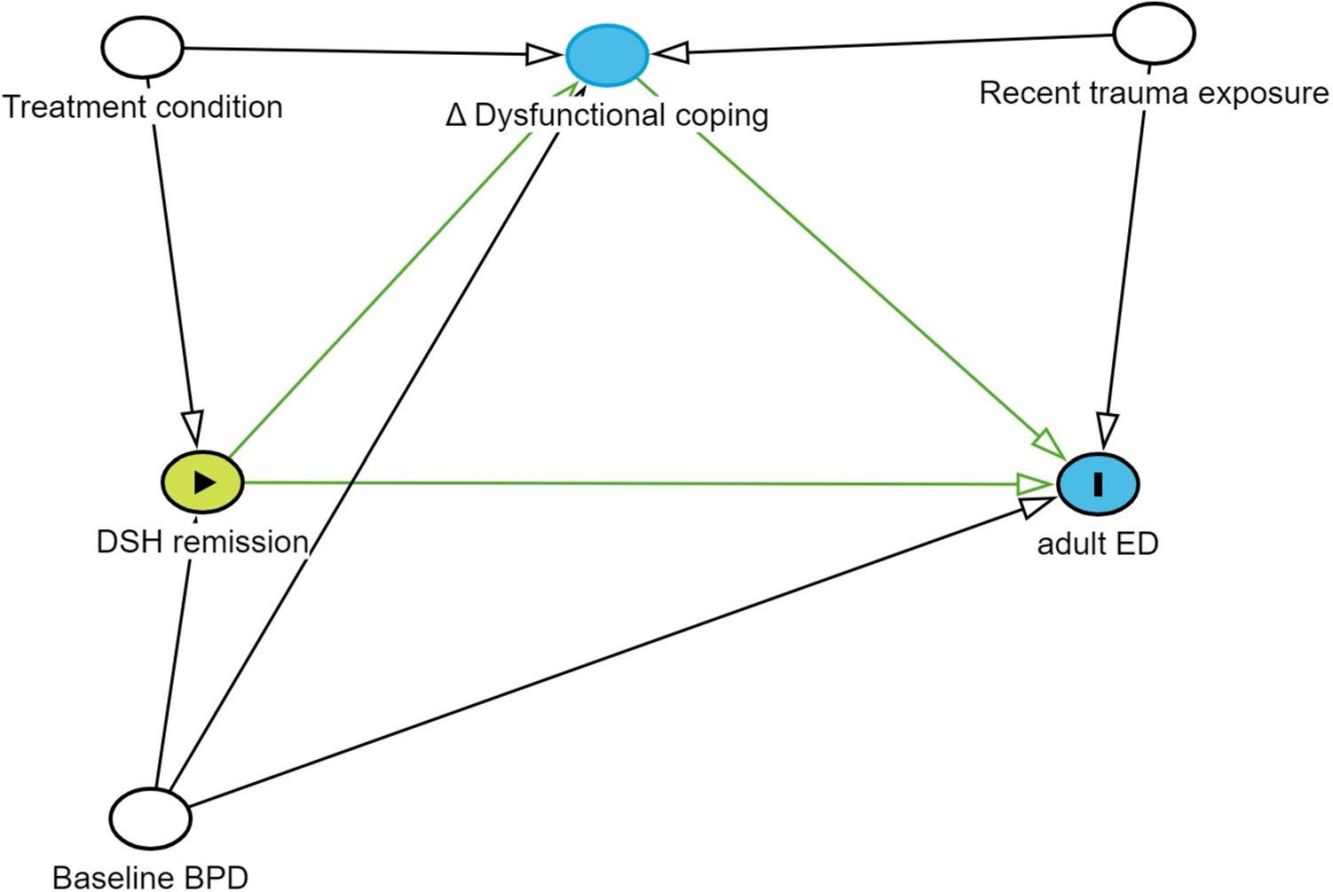
Directed acyclic graphs illustrating the causal mediation model

See Fig. 2 for an overview over the assumed causal relationships. DSH remission was assessed 1.6 years post baseline (1 year after treatment completion), reductions in dysfunctional coping were calculated as the difference between the 3.1 year follow-up and the 12.4 year follow-up, and ED was measured at the 12.4 year follow-up. Missing at random was assumed, and there were no associations between missing values and either the exposure, outcome, mediator or confounders.

Model fit checking and selection were done in separate multivariate linear regression models for both the outcome and mediator before these were combined to estimate direct and indirect effects. In this initial process, potential interaction effects between the variables were tested, and models were conducted with and without the confounding variables.

## Results

### Sample characteristics

The final sample consisted of 61 (of 77) participants with a mean age of 28.1 (baseline 15.6) and where the majority were female and of Norwegian ethnicity (both 87%). All sample characteristics are summarized in Table 1, further details can be seen in the primary outcomes study (Mehlum et al. under review).

**Table 1:** Sample characteristics at the time of follow-up in a 12.4 years follow-up assessment of adolescents included in a randomized controlled trial comparing dialectical behaviour therapy or enhanced usual care

Variable	DBT (n = 32)	EUC (n = 29)	Total (N = 61)
Age, mean (SD)	28.5 (1.8)	27.6 (2.0)	28.1 (1.9)
Gender, n (%)			
Female	28 (87)	25 (86)	53 (86)
Norwegian ethnicity	28 (87)	25 (86)	53 (86)
Education level, n (%)			
Secondary education	3 (9)	6 (20)	9 (14)
High school	11 (34)	10 (34)	21 (34)
Some higher education	2 (6)	0	2 (3)
Undergraduate	11 (34)	10 (34)	21 (34)
Graduate	5 (15)	3 (10)	8 (13)
Occupational status, n (%)			
Full-time employment/studies	18 (56)	20 (68)	38 (62)
Part time employment/studies	6 (18)	3 (10)	9 (14)
Unemployed	2 (6)	1 (3)	3 (4)
Disability > 50%	4 (12)	4 (13)	8 (13)
Maternity/paternity leave	2 (6)	1 (3)	3 (4)
Marital status, n (%)			
Single	4 (12)	9 (31)	13 (21)
Partner	6 (18)	6 (20)	12 (19)
Married/living with partner	21 (65)	14 (48)	35 (57)
Divorced	1 (3)	0	1 (1)

Note: Decimals not shown. All percentages add up to 100% when accounting for decimals

### Use of functional and dysfunctional coping strategies over time by treatment condition

In the DBT-A group, there was a statistically significant increase in functional coping over time (t = −2.29, p = 0.03) as well as a significant decrease in dysfunctional coping (t = 2.71, p = 0.01). Comparably, the EUC group also showed a statistically significant increase in functional coping (t = −2.29, p = 0.03), however, no change was observed in dysfunctional coping. There were no statistically significant differences between the groups.

### ED in adulthood

The mean score of self-reported ED (DERS-16) in the whole sample at the 12.4 year follow-up was 41.9 (SD = 15.27). Mean DERS scores were 41 (SD = 13.90) in the DBT-A group and 42.86 (SD = 16.79) in the EUC group, and this difference was not statistically significant.

### Mediation analysis: Direct and indirect effects of early DSH remission on adult ED

The mediation analysis included two regression models; one was fitted for the outcome (ED in adulthood, see Table 2) and a second for the mediator (changes in dysfunctinoal coping, see Table 3). Both DSH remission (the exposure; β = −13.20, p = 0.018) and dysfunctional coping reduction (the mediator; β = −19.90, p = 0.001) showed strong, negative associations with the outcome. Moreover, there was a statistically significant interaction between the two (β = 39.78, p = 0.001). Neither treatment condition nor a diagnosis of BPD at baseline showed any statistically significant association with the outcome, while number of exposures to physical abuse trauma in the final follow-up interval was positively associated with increased adult ED (β = 0.33, p = 0.018). The confounding variable *total number of traumatic episodes* was statistically significant, however when we tested whether there were differences between the subscales of “sexual abuse”, “physical abuse” and “other” types of trauma, the only statistically significant of these was "physical abuse". When combining the total number and physical abuse only, the effect was stronger for physical abuse, so we decided to retain that in the model and remove the total number to avoid duplication of data. Inclusion of this confounder did not affect estimates substantially, however improved model fit. Both DSH remission (0.26, p = 0.069) and DBT-A (0.24, p = 0.068) was positively associated with the mediator, however these associations did not reach statistical significance (see Table 3).

**Table 2:** Multivariate regression coefficients for the outcome model (adult ED)

Emotion dysregulation	β	SE	p-value	CI 95%
DSH remission	**−13.20**	**5.32**	**.018**	**−23.97–2.43**
Δ dysfunctional coping (dyscop)	**−19.90**	**5.71**	**.001**	**−31.18–8.62**
DSH rem* Δdyscop	**39.78**	**11.35**	**.001**	**16.81–62.76**
Treatment condition	**−**.61	4.29	.89	**−**9.29**–**8.07
Trauma episodes (violence)	**.33**	**.13**	**.018**	**.059–.59**
BPD at baseline	1.63	5.78	.78	**−**10.07**–**13.34
_cons	42.96	7.24	.000	28.30**–**57.62

Bold values reflect statistically significant estimates at the 0.05 level

**Table 3:** Multivariate regression coefficients for the mediator (reduction in dysfunctional coping) model

Δ dysfunctional coping	β	SE	p-value	CI 95%
DSH remission	.26	.14	.069	**−**.02**–**.55
Treatment condition	**−**.24	.13	.068	**−**.49**–**.02
Trauma episodes (violence)	**−**.004	.004	.29	**−**.01**–**.004
BPD at baseline	**−**.16	.18	.38	**−**.53**–**.20

Causal inference effect estimates were calculcated both as marginal and conditional on treatment condition, as displayed in Table 4. In the marginal model, there was a strong pure natural direct effect (PNDE) (−14.05, p = 0.024) of DSH remission on adult ED, which in essence is the effect of DSH remission when the level of dysfunctional coping is set to the level it would have been in the absence of DSH remission. When estimating the conditional effects (on treatment condition), the PNDE was even stronger in the EUC group (−24.00, p = 0.02), and non-significant in the DBT-A group (−14.56, p = 0.08). None of the indirect effect estimates of DSH remission on ED through reduction in dysfunctional coping were statistically significant, suggesting that the effect of DSH remission is not mediated by changes in dysfunctional coping. The total effect is the sum of natural direct and indirect effects, and these results suggest the presence of a direct effect of DSH remission on ED. There was a strong, negative total conditional effect of DSH remission on adult ED in the EUC group (−18.78, p = 0.05), while the total effect did not reach statistical significance in neither the DBT-A group (−9.34, p = 0.25) nor in the marginal model (−8.83, p = 0.09). In sum, DSH remission one year after treatment was highly associated with adult ED in both the marginal and conditional models. Thus, lack of DSH remission is both associated with increased risk for adult ED in general, and participants who received EUC and did not achieve DSH remission within the first year after treatment were particularly at risk for higher ED in adulthood.

**Table 4:** Causal effect estimates for marginal and conditioned effects

	**Marginal**		**Conditional (EUC)**		**Conditional (DBT-A)**	
**Effect estimate**	Estimate (SE)	p-value (CI95%)	Estimate (SE)	p-value (CI95%)	Estimate (SE)	p-value (CI95%)
Controlled direct	**−**8.42 (4.81)	.08 (**−**17–1.00)				
Pure natural direct	**−14.05 (6.22)**	**.024 (−26.25–1.85)**	**−24.00 (10.21)**	**.02 (−44.02–3.99)**	**−**14.56 (8.39)	.08 (**−**31.01–1.89)
Pure natural indirect	**−**5.23 (3.16)	.09 (**−**11.42**–**.97)	**−**5.23 (3.16)	.09 (**−**11.42**–**.97)	**−**5.23 (3.16)	.09 (**−**11.42**–**.97)
Total natural direct	**−**3.60 (6.54)	.58 (**−**16.41–9.21)	**−**13.55 (10.84)	.21 (**−**34.79–7.69)	**−**4.11 (9.48)	.67 (**−**22.68**–** **−**14.46)
Total natural indirect	5.22 (3.88)	.18 (**−**2.38–12.83)	5.22 (6.21)	.40 (**−**6.95–17.40)	5.22 (6.21)	.40 (**−**6.95–17.40)
Total	**−**8.83 (4.81)	.09 (**−**19.09–1.43)	**−18.78 (9.62)**	**.05 (−37.65–.08)**	**−**9.34 (8.16)	.25 (**−**25.32–6.65)

Bold values reflect statistically significant estimates at the 0.05 level

### What specific behaviors matter? A post-hoc analysis of specific coping strategies’ effect on ED

Although dysfunctional coping did not mediate the association between DSH remission and adult ED, there was a strong association between dysfunctional coping strategies use during adolescence and subsequent adult ED, so we wanted to explore whether specific dysfunctional strategies were more strongly related to this outcome than others. In a series of post-hoc univariate linear regression models, more frequent use of the following coping strategies measured in adolescence was significantly associated with higher levels of ED in adulthood: *“Figured out who to blame”* (β = 5.31, p = 0.032*), “Took it out on others”* (β = 4.54, p = 0.037)*, “Blamed others”* (β = 6.82, p = 0.027)*, “Wished that I could change the way I felt”* (β = 5.64, p = 0.014)*, “Felt bad that I couldn’t avoid the problem”* (β = 4.65, p = 0.027) and *“Wish that I could change what had happened”* (β = 6.88, p = 0.001).

## Discussion

The present study demonstrated that in adolescents with BPD features and repeated DSH, subjects who had achieved DSH remission within 1 year after treatment completion displayed significantly lower levels of ED as adults on average 10.8 years later. This effect was not mediated through changes in use of dysfunctional coping strategies and was particularly robust for participants who did not receive DBT-A. Moreover, while participants who received DBT-A reported reductions in dysfunctional coping over time, there was no difference in the EUC group. This is the first study of this high-risk patient group of adolescents with follow-up assessments in adulthood, as well as the first to demonstrate a direct effect of DSH remission to lower adult ED. These results provide new insight into the long-term relationship between DSH and ED, and highlights the importance of early DSH remission.

Our main aim was to investigate whether ER capacity could be predicted by early behavioural change in adolescents treated for repeated DSH. At the final follow-up, most participants in this study no longer reported DSH. Accordingly, although rates of DSH naturally decrease into adulthood [58], our results could indicate the importance of achieving DSH remission one year after treatment in adolescence in relation to adult life capacity to regulate emotions effectively a whole decade later – thus that *early* remission matters. These findings suggest DSH remission as a prognostic factor in reducing BPD pathology in the long-term [59], and might reflect how repeated DSH can reinforce ED as opposed to managing to defer from self-harm despite urges [35]. Notably, this effect was particularly strong in EUC and not statistically significant when conditioning on DBT-A. There are various potential explanations for this. Firstly, although DSH remission did not vary significantly between the groups, mean frequency of DSH certainly did. We have previously reported a statistically significant difference between the groups, favouring DBT-A [43; M = 5.5, as opposed to M = 14.8 in the EUC group]. Thus, the difference between remission and not might not be as meaningful in the DBT-A group, since the level of DSH was generally lower in that group. In essence, this might imply that few DBT-A participants continue with repeated DSH compared to the EUC group. Accordingly, the contrast between remission or not is naturally larger in the EUC group, which perhaps imply that more pervasive patterns of *repeated* DSH is what predicts adult ED. In contrast, the difference between zero and a few DSH episodes (as reported in the DBT-A group) might not be as predictive. Clinically, this makes sense as there is a difference between sporadic instances of DSH and pervasive, repeated patterns, where the latter is more associated with ED [60]. Thus, the direct effect of DSH remission on adult ED might be explained similarly to removing a risk factor [61], in effect a consequence of the removal of the reinforcing influence that repeated DSH would otherwise likely have on ED [10, 28]. Secondly, there might be additional pathways through which DBT-A influence ED, that are not investigated in the present analysis. Finally, there might be [unknown] unmeasured confounding variables that influenced the association. Nevertheless, these results highlight the importance of early DSH remission in adolescents, and how it can have long-lasting implications for adult emotional health.

In this study, both early remission of DSH and lower use of dysfunctional coping strategies in adolescence was associated with improved long-term emotion regulation capacity. Whereas on the whole, most participants increased functional coping from adolescence through adulthood, only those who received DBT-A reported reduced dysfunctional coping over time. The association between dysfunctional coping in adolescence and ED in adulthood, extends the litterature showing that DBT is superior in reducing dysfunctional coping strategies in adults [62], and is concordant with Southward et al.’s [37] findings that such changes predicted less ED in adults who received DBT. Of note, most mechanism-of-change studies investigating coping focus on DSH as the outcome, and not ED itself. In this study, DSH remission was the exposure rather than the outcome, illustrating the importance of *early* change as a possible mechanism of change in treatment of adolescent ED.

Interestingly, the six coping strategies that were associated with higher levels of ED, could thematically be grouped into two factors; “blaming others”, and “non-acceptance”. This is in line with previous studies that have found associations and even a mediating effect with “acceptance without judgment”, both when it comes to general psychopathology [23] and DSH specifically [21, 22]. To our knowledge, a link between “blaming others” and ED has not formerly been reported, however, Kramer et al. [63] discuss how *rejecting* anger (defined as “a reactive emotional state, aiming at getting rid of a particular content, by accusing or blaming the other”) might impede adaptive emotional processing and need longer, more intensive treatments to be resolved in BPD patients.

Although the main focus of this study was the role of behavioural patterns’ effect on emotion regulation development, it is clearly important to consider other factors as well, including but not limited to, the social context, important life events as well as other therapeutic processes known to affect BPD treatment response. In this sample, having experienced more physical abuse was associated with more severe ED, which brings attention to the consequences that physical abuse can have for emotion regulation development and the importance of investigating extratherapeutic factors. Moreover, it is likely that other [unmeasured] therapuetic processes besides behavioural change have had an effect on ED, e.g. reflective functioning [64].

### Clinical implications

The interconnected relationship between DSH and ED is well-known, as is the tendency for DSH to cease in adulthood, which is also notable in the present sample. Thus, the novelty in these findings is not that reduced DSH predicts ED, however the timing at which it happens. The findings in this study illustrates the importance of early intervention, and highlights how focusing specifically on early behavioural change can lead to changes in a general pattern like ED over a decade later. This is important as ED in adulthood is associated with adverse mental health outcomes as well as poorer functioning across a number of domains. Arguably, our results suggest that the long-term interplay between ED and DSH might be a potential mechanism of long-term change. In DBT-A, the first priority in the treatment is to reduce life-threathening behavior such as DSH, before working on other treatment goals. Notably, that the effect of remission was particularly strong in the non-DBT group highlight the importance of increased accesibility to effective treatments in terms of DSH reduction. In sum, this study highlights the importance of directly and explicitly targeting DSH remission *early* in treatment. These results indicate that treatment should focus on reducing self-harm and offer alternative coping strategies.

### Limitations and future directions

This study benefitted from a unique sample of treatment-seeking adolescents in a real-world, multicentre health care setting, followed for over 12 years into adulthood with both clinical and self-reported data. Moreover, it was a prospective long-term follow-up design of an RCT and, despite the very long follow-up interval, we had a high retention rate (80%). However, there are also several limitations to the study. First, the sample size is small, which might possibly have been too small to detect more nuanced differences, and results should thus be interpreted with caution due to the lack of an a priori power calculation. Second, the length of the final follow-up period of over 9 years substantially increases risk of unmeasured confounding, for example related to type of treatment received during this time. Third, the sample was demographically homogenous, and the findings are not generalizable to boys or minority youth. Moreover, the main outcome in this study was only measured at one timepoint and not at baseline, which impeded longitudinal analyses of treatment*time effects, and also the possibility to investigate changes occuring during the course of treatment. Future studies should aim to explore potential long-term mechanisms of therapeutic change in crossover or parallel designs, where potential mediators could be manipulated experimentally. Furthermore, replication studies or adult follow-up assessments of other DSH treatments for adolescents, such as emotion regulation individual therapy for adolescents [65] or the Cutting Down Program [66], are warranted. The long-term consequences of dysfunctional coping strategies could be further investigated, including potential substitution of DSH with other functionally equivalent problem behaviour, such as substance abuse or eating problems. Finally, ED, DSH, coping strategies and their interplay should be investigated with technological advances such as ecological momentary assessment, as this is more suitable to capture these variables’ dynamic, interacting and fluctuating nature.

## Conclusions

This study demonstrated the importance of early remission of DSH in adolescence, when it comes to long-term emotion regulation capacity in adulthood – almost a decade later. Higher use of dysfunctional coping strategies and having experienced recent physical abuse were also associated with increased ED in adulthood. Access to evidence-based treatment in adolescence can have far-reaching implications for adult mental health.

## Data Availability

Unfortunately, we are not able to publish raw data in this study. This study uses person sensitive data, related to information regarding participant’s mental health, suicidality and functioning. All participants have signed informed consent to the data being analyzed and that the results of these analyses could be published in the scientific literature. However, participants in our study have not consented to raw data being made available. To protect this highly sensitive information was considered an important ethical aspect in this study.

## Abbreviations

DBT-A: Dialectical behaviour therapy adapted for adolescents
RCT: Randomized controlled trial
EUC: Enhanced usual care
DSH: Deliberate self-harm
ED: Emotion dysregulation
